# Efficacy of Semi-Solid Nutrients on Gastroesophageal Reflux Symptoms in Neurologically Impaired Patients Evaluated Using Multichannel Intraluminal Impedance-pH Monitoring: A Report of Two Cases

**DOI:** 10.7759/cureus.96446

**Published:** 2025-11-09

**Authors:** Daisuke Masui, Naoki Hashizume, Yoshinori Koga, Saki Sakamoto, Tatsuru Kaji

**Affiliations:** 1 Pediatric Surgery, Kurume University School of Medicine, Kurume, JPN; 2 Pediatric Surgery, Kurume University School of Medicine, Fukuoka, JPN

**Keywords:** gastroesophageal reflux, liquid nutrition, multichannel intraluminal impedance-ph monitoring, neurologically impaired patient, semi-solid nutrition

## Abstract

Gastroesophageal reflux (GER) is a common and serious complication in patients receiving enteral nutrition, which can make continuing enteral feeding difficult. Semi-solid enteral nutrition confers the advantage of reducing the risk of diarrhea and GER; however, despite its widespread use in Japan, limited published data are available on this method. Here, we report two neurologically impaired patients whose GER symptoms improved with semi-solid nutrient administration. Multichannel intraluminal impedance-pH (MII-pH) monitoring was used to evaluate GER progression. The first patient, a 47-year-old man with severe scoliosis and a history of endoscopic submucosal resection for cardioesophageal cancer, was referred to our hospital for vomiting. He was treated with a potassium-competitive acid blocker and semi-solid nutrients (RACOL®-NF Semi-Solid for Enteral Use, EN Otsuka Pharmaceutical Co., Ltd., Hanamaki, Japan), which helped manage GER episodes. The second patient, a 9-year-old boy with repeated aspiration pneumonia, was referred for vomiting. MII-pH monitoring showed that bolus infusion of liquid nutrients by his caregiver aggravated his GER. However, switching to semi-solid nutrients alleviated his GER symptoms. These case studies suggested that semi-solid nutrition may be a useful alternative for managing GER symptoms in patients experiencing difficulties with liquid nutrition.

## Introduction

Neurologically impaired patients often present with physical manifestations, such as scoliosis, which can lead to vomiting and severe aspiration pneumonia due to gastroesophageal reflux (GER). In particular, liquid nutrients can cause GER, making it challenging to continue enteral nutrition.

Semi-solid nutrients confer the advantage of lowering the risk of diarrhea and GER. Semi-solid nutrients are widely used in Japan and were developed as an alternative to standard liquid enteral nutrients to help prevent feeding-related GER [1,2]. However, despite their widespread use in Japan, there is limited published research on this feeding method.

Multichannel intraluminal impedance-pH (MII-pH) monitoring has been proposed as a novel technique with the ability to detect all types of reflux and evaluate the relationship between symptoms and both acid and non-acid refluxes [3].

Here, we present two cases where in the administration of semi-solid nutrients improved GER symptoms in patients undergoing MII-pH monitoring.

## Case presentation

Case 1

**Figure 1:**
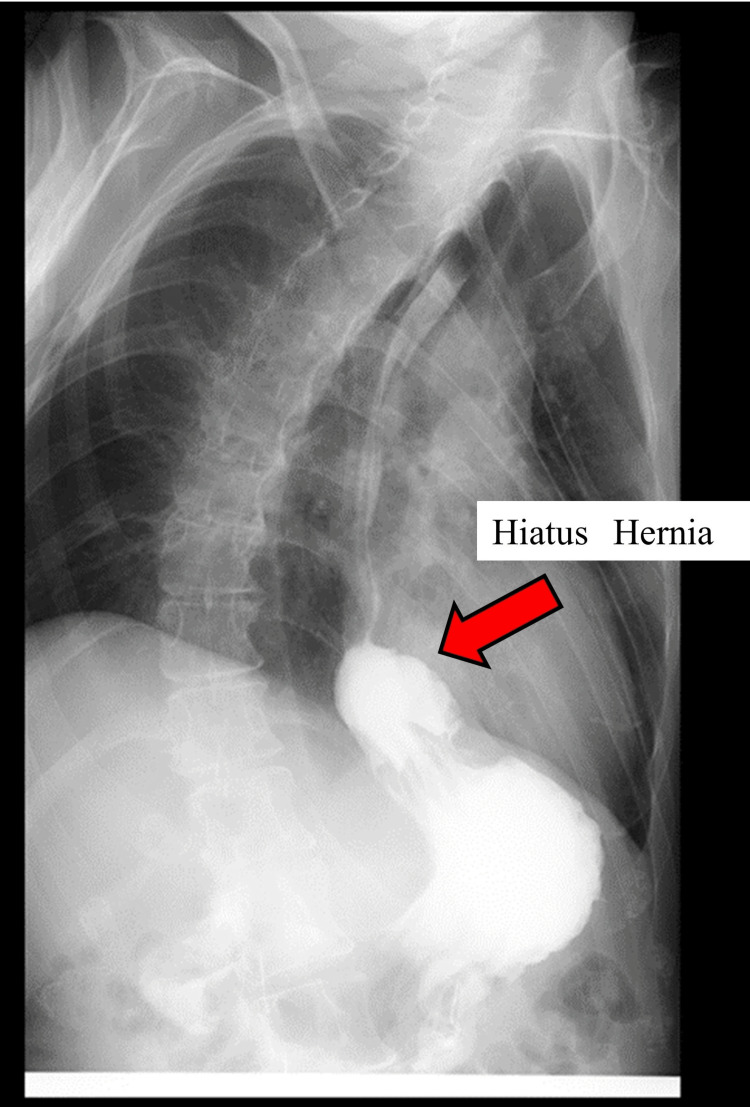
An upper gastrointestinal (GI) contrast image series revealing a sliding hiatal hernia (red arrow).

A 47-year-old man diagnosed with spastic cerebral palsy was referred to our hospital for vomiting. He also exhibited refractory epilepsy, global developmental delay. His medical history revealed that he had undergone endoscopic mucosal resection (EMR) for cardioesophageal cancer. He vomited two or three times a day. His gastric juice was dark brown while on a potassium-competitive acid blocker (P-CAB). He also lost weight. Semi-digestive liquid nutrients were used through the gastrostomy tube, and the speed of administration was 150 mL/hour under continuous feeding. An upper gastrointestinal (GI) contrast image series revealed a sliding hiatal hernia (Figure 1).

**Figure 2:**
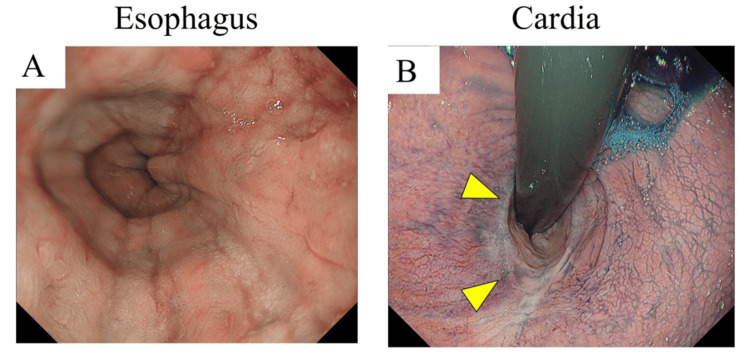
Endoscopic findings. (A) Endoscopy shows Los Angeles grade M esophagitis. (B) A post-EMR scar (yellow triangle) is located on the cardia. EMR, endoscopic mucosal resection

Endoscopy revealed Los Angeles grade M esophagitis and a post-EMR scar (Figure 2).

**Figure 3:**
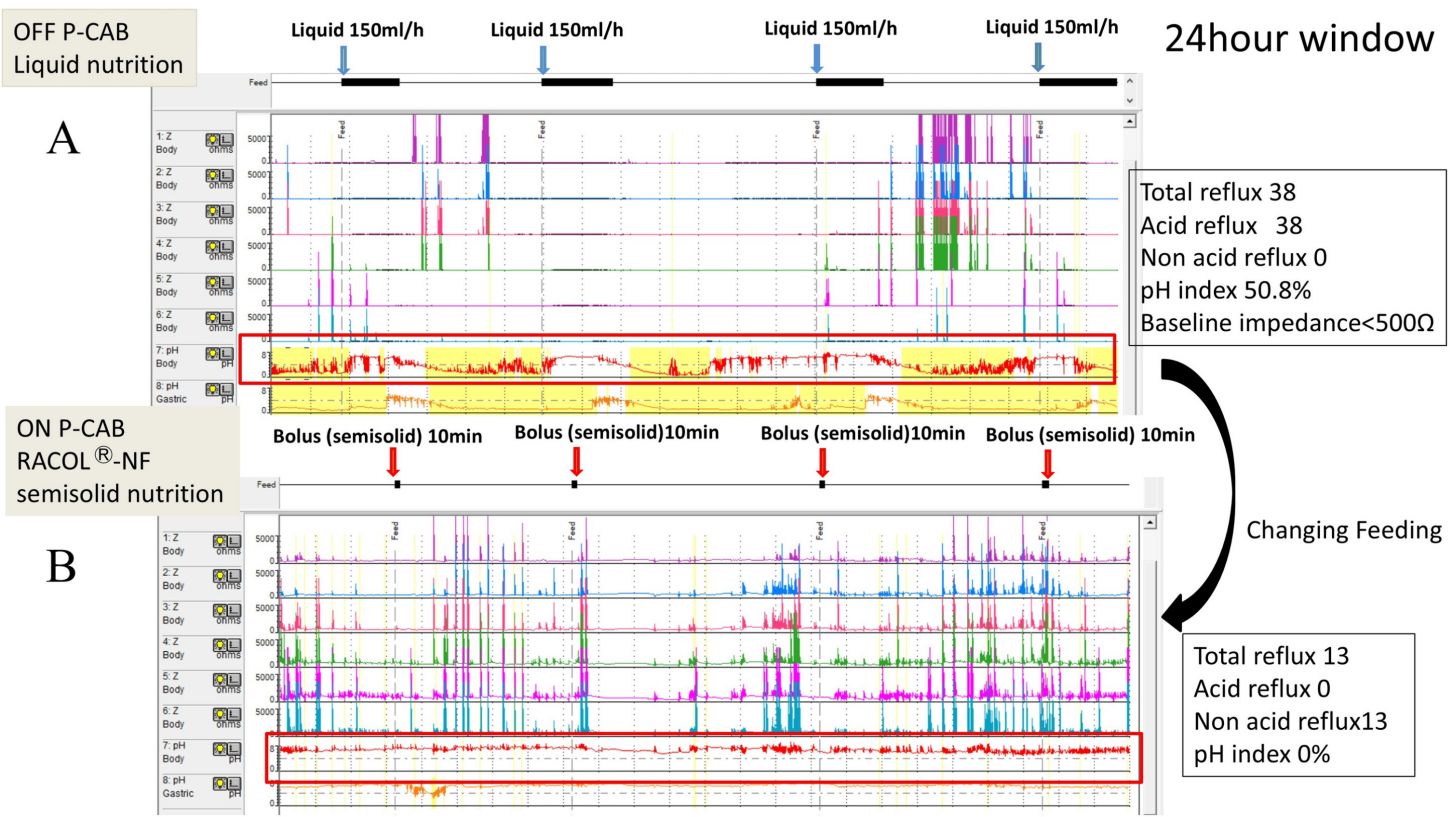
MII-pH monitoring findings (24-hour window). (A) MII-pH monitoring showed an acid exposure time of 50.8% and a baseline impedance below 500 Ω under liquid nutrition. Liquid nutrients were administered at 150 mL/hour, four times per day (blue arrows). The total reflux events, acid reflux events, and non-acid reflux events were 38, 38, and 0, respectively. The patient was unable to undergo fundoplication due to severe scoliosis and a post-EMR condition following gastric cancer. (B) The patient was subsequently treated with a potassium-competitive acid blocker (P-CAB) and semi-solid nutrients (RACOL®-NF Semi-Solid for Enteral Use, EN Otsuka Pharmaceutical Co., Ltd., Hanamaki, Japan). Semi-solid nutrients were administered over 10 minutes, four times per day, by bolus feeding (red arrows). After treatment, MII-pH monitoring showed an acid exposure time of 0% and marked improvement in gastroesophageal reflux symptoms. The total reflux events, acid reflux events, and non-acid reflux events were 13, 0, and 13, respectively. MII-pH, multichannel intraluminal impedance-pH; EMR, endoscopic mucosal resection

MII-pH monitoring performed during proton pump inhibitor therapy showed acid exposure time of 50.8% and baseline impedance below 500 Ω (Figure 3). Also, total reflux events, acid reflux events, and non-acid reflux events were 38, 38, and 0. This patient was treated with a P-CAB and semi-solid nutrients (RACOL®-NF Semi-Solid for Enteral Use, EN Otsuka Pharmaceutical Co., Ltd., Hanamaki, Japan) due to severe scoliosis and his post-EMR condition. The patient’s course was uneventful while receiving semi-solid nutrients. Also, the symptoms improved immediately after the change in nutrition. After treatment, MII-pH monitoring showed acid exposure time of 0% and marked GER symptom improvement (Figure 3).

Also, the total reflux events, acid reflux events, and non-acid reflux events were 13,0, and 13. After three years of follow-up, the patient continues to show no recurrence of symptoms.

Case 2

**Figure 4:**
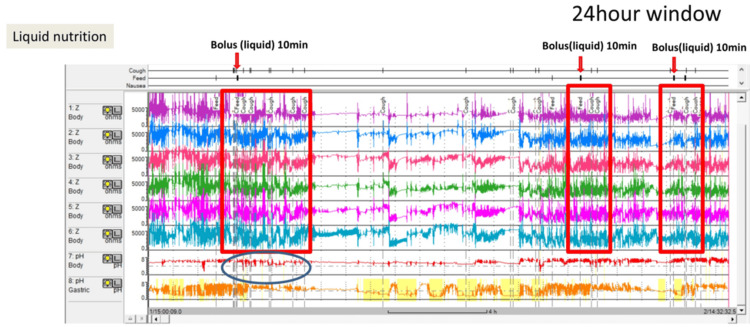
MII-pH monitoring (24-hour window). MII-pH monitoring revealed an acid exposure time of 0.2%. The total reflux events, acid reflux events, non-acid reflux events, and proximal reflux events were 25, 5, 20, and 11, respectively. Liquid nutrition was administered over 10 minutes, three times per day, by bolus feeding (red arrow). Although continuous feeding is generally recommended for liquid nutrition, MII-pH monitoring indicated that the caregiver had been administering liquid nutrition as bolus injections. MII-pH, multichannel intraluminal impedance-pH

A nine-year-old boy with spastic cerebral palsy following cytomegaloviral encephalitis was referred to our hospital for evaluation of vomiting. He exhibited refractory epilepsy, global developmental delay. Semi-digestible liquid nutrients were administered through a gastrostomy tube; however, the method of administration by the caregiver was unknown. His medical history revealed recurrent episodes of pneumonia. Evaluation of swallowing revealed no abnormalities. However, he continued to develop recurrent fevers and vomited each time liquid nutrition was administered. Also, he occasionally experienced desaturation events due to excessive secretion. Therefore, the patient was evaluated for gastroesophageal reflux disease (GERD). An upper gastrointestinal contrast study showed one reflux episode during administration of the contrast agent. MII-pH monitoring revealed an acid exposure time of 0.2%. The total reflux events, acid reflux events, non-acid reflux events, and proximal reflux events were 25, 5, 20, and 11, respectively (Figure 4).

**Figure 5:**
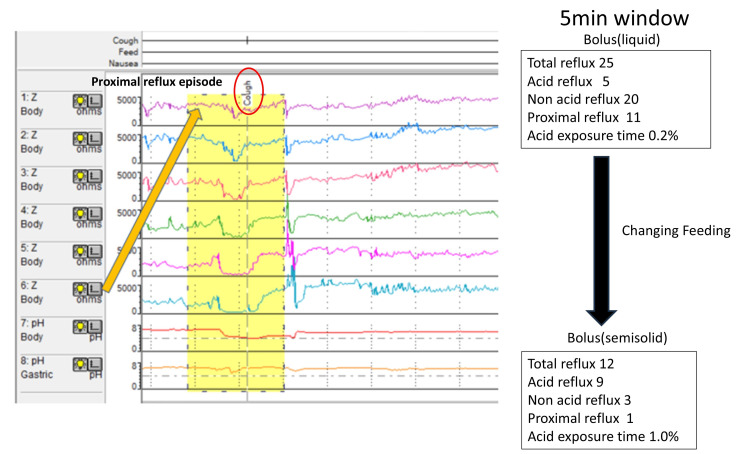
MII-pH monitoring (5-minute window). Eleven of the proximal reflux episodes were associated with coughing. Findings from MII-pH monitoring and the medical record indicated that these proximal reflux episodes were triggered by bolus infusion of liquid nutrients administered by the caregiver. Semi-solid nutrients were administered over 10 minutes, three times per day, by bolus feeding. The patient’s clinical course was uneventful under semi-solid nutrient administration. After treatment, symptoms such as vomiting and excessive secretions during feeding improved immediately. The number of proximal reflux events decreased from 11 to 1. MII-pH, multichannel intraluminal impedance-pH

Notably, 11 of these episodes were proximal reflux episodes associated with coughing (Figure 5).

MII-pH monitoring and medical record findings indicated that these proximal reflux episodes were triggered by the bolus infusion of liquid nutrients by the caregiver. Although continuous feeding is recommended for liquid nutrition, MII-pH monitoring revealed that the caregiver administered liquid nutrition as bolus injections. The patient was then treated via semi-solid nutrient (RACOL®-NF Semi Solid for Enteral Use) administration. Semi-solid nutrient was administered for 10 minutes, three times a day by bolus feeding. The patient was uneventful under semi-solid nutrients. After treatment, symptoms such as vomiting and excessive secretions during feeding improved. MII-pH monitoring revealed an acid exposure time of 1.0%. Also, total reflux events, acid reflux events, non-acid reflux events, and proximal reflux events were 12,9, 3, and 1. Proximal reflux events decreased from 11 to 1. The frequency of hospital admissions has reduced. After three years of follow-up, the patient continues to show no recurrence of symptoms.

## Discussion

GER is a common and serious complication in patients receiving enteral nutrition, which can make it difficult to continue this type of feeding. Our case studies demonstrated that switching to semi-solid nutrients allowed continuation of enteral nutrition in patients who struggled with GER symptoms. In an animal model of GERD, semi-solid feeding significantly reduced the frequency of GER during and after feeding periods [4]. To date, three studies have assessed GER in patients receiving semi-solid nutrients [2,5,6]. A meta-analysis showed that enteral tube feeding with semi-solid nutrients significantly lowered the prevalence of GER [7].

In our cases, RACOL®-NF Semi-Solid for Enteral Use was used. Its viscous agents are alginic acid and powdered agar. In Japan, some semi-solid nutrients are designed such that they can change form: they are liquid when administered through the tube, semi-solidify in the stomach, and then return to a liquid state in the intestine [8,9]. Because this form change depends on pH, the transition from liquid to semi-solid may not be smooth when gastric acid suppressants are used. In our first case study, RACOL®-NF Semi-Solid for Enteral Use helped manage GER symptoms even when the patient was on P-CAB treatment. Additionally, this semi-solid enteral nutrient is less expensive than other semi-solid feeding options and is a suitable choice for patients who cannot undergo fundoplication. In our second case study, the patient experienced GER primarily due to bolus infusions of liquid nutrients by the caregiver; however, switching to semi-solid nutrients reduced vomiting and secretions.

Adaptive relaxation modulates gastric tone in response to certain meal properties, supports physiological movement within the stomach [10], and is an important factor related to the viscosity of enteral nutrients. Recently developed semi-solid enteral nutrients are more likely to induce adaptive relaxation than liquid enteral nutrients and thus can potentially suppress GER.

Various methods have been used to evaluate GER. In some studies, intragastric and esophageal distribution were tracked using a scintillation camera. In this approach, the reflux of contrast agent into the esophagus was observed by radiographic examination. Thirty minutes after the administration of the contrast agent, a computed tomography scan of the esophagus was performed with 1 cm slice thickness, and GER was diagnosed if the Hounsfield number in each slice was >100 [2]. The upper GI tract was also examined radiologically from the start of administration to 1 min after completion of administration of the contrast agent. GER was diagnosed if any reflux of contrast agent into the esophagus was detected [6]. Nishiwaki et al. defined the GER index as the maximum percentage of esophageal radioactivity count relative to total infused radioactivity. Their study involving 30 participants reported that semi-solid nutrients significantly reduced the GER index [5]. Two studies classified GER simply as present or absent [2,6]. To the best of our knowledge, our case report is the first to assess the effectiveness of semi-solid nutrition for GER using MII-pH monitoring. Since MII-pH can detect non-acid reflux, reflux episodes can be assessed under acid suppression therapy regardless of the type of diet. Furthermore, unlike the upper GI contrast image series, which has a limited testing window, the MII-pH test involves 24-hour monitoring, allowing assessment under various conditions, regardless of meal or activity times.

In our first case, reflux episodes were assessed during acid suppression, and the combination of semi-solid nutrients and P-CAB proved effective. In the second case, the MII-pH test showed that bolus administration of liquid nutrients was performed by the caregiver. Thus, MII-pH facilitates objective evaluation of the underlying pathophysiology.

This case report has several limitations. First, the report includes only two cases, which limits the generalizability of the findings. Therefore, larger controlled studies are needed to validate the efficacy and safety of this treatment. Second, patient-specific factors, including the severity of neurological impairment and the feeding technique used, may have influenced the outcomes and could act as potential confounders. Finally, although our cases observed three years after changing feeding, long-term outcomes and the sustainability of the intervention were not assessed. Therefore, the durability of the observed benefits remains uncertain.

## Conclusions

In conclusion, two cases highlight that the administration of semi-solid nutrients improved GER symptoms in patients undergoing MII-pH monitoring. Semi-solid nutrition may represent a potential option for managing GER in patients who are intolerant to liquid nutrition. Further larger controlled studies are needed to confirm the efficacy and safety of semi-solid nutrients.
